# Human Blood Serum Induces p38-MAPK- and Hsp27-Dependent Migration Dynamics of Adult Human Cardiac Stem Cells: Single-Cell Analysis via a Microfluidic-Based Cultivation Platform

**DOI:** 10.3390/biology10080708

**Published:** 2021-07-24

**Authors:** Anna L. Höving, Julian Schmitz, Kazuko E. Schmidt, Johannes F. W. Greiner, Cornelius Knabbe, Barbara Kaltschmidt, Alexander Grünberger, Christian Kaltschmidt

**Affiliations:** 1 Department of Cell Biology, Faculty of Biology, Bielefeld University, 33615 Bielefeld, Germany; kazuko_elena.schmidt1@uni-bielefeld.de (K.E.S.); Johannes.Greiner@uni-bielefeld.de (J.F.W.G.); Barbara.Kaltschmidt@uni-bielefeld.de (B.K.); C.Kaltschmidt@uni-bielefeld.de (C.K.); 2 Heart and Diabetes Centre NRW, Institute for Laboratory and Transfusion Medicine, Ruhr-University Bochum, 32545 Bad Oeynhausen, Germany; cknabbe@hdz-nrw.de; 3 Multiscale Bioengineering, Faculty of Technology, Bielefeld University, 33615 Bielefeld, Germany; j.schmitz@uni-bielefeld.de (J.S.); alexander.gruenberger@uni-bielefeld.de (A.G.); 4 Molecular Neurobiology, Faculty of Biology, Bielefeld University, 33615 Bielefeld, Germany

**Keywords:** human cardiac stem cells, single-cell analysis, p38-MAPK, Hsp27, cell morphology, stem cell migration, microfluidics, human blood serum

## Abstract

**Simple Summary:**

Adult human stem cells possess the ability to contribute to endogenous regeneration processes of injured tissue by migrating to specific locations. For stem cell-based clinical applications it is highly important to gain knowledge about the migration behavior of adult human stem cells and the underlying molecular mechanisms of this ability. Human blood serum has been shown to have beneficial effects on other regenerative capacities of adult human stem cells. Within this study we tested the effect of human blood serum on the migration behavior of stem cells from the human heart. We used a microfluidic cultivation device, which allowed us to monitor the living cells and their movement behavior in real time. After addition of human blood serum, the heart stem cells increased their speed of movement and covered distance. Further, we observed that this effect could be diminished by inhibition of a specific kinase, p38-MAPK. Thus, our data suggest beneficial effects of human blood serum on adult human heart stem cells dependent on p38-MAPK. Our study contributes to a deeper understanding of the dynamics of stem cell migration and introduces a new platform to monitor stem cell movement in real time.

**Abstract:**

Migratory capabilities of adult human stem cells are vital for assuring endogenous tissue regeneration and stem cell-based clinical applications. Although human blood serum has been shown to be beneficial for cell migration and proliferation, little is known about its impact on the migratory behavior of cardiac stem cells and underlying signaling pathways. Within this study, we investigated the effects of human blood serum on primary human cardiac stem cells (hCSCs) from the adult heart auricle. On a technical level, we took advantage of a microfluidic cultivation platform, which allowed us to characterize cell morphologies and track migration of single hCSCs via live cell imaging over a period of up to 48 h. Our findings showed a significantly increased migration distance and speed of hCSCs after treatment with human serum compared to control. Exposure of blood serum-stimulated hCSCs to the p38 mitogen-activated protein kinase (p38-MAPK) inhibitor SB239063 resulted in significantly decreased migration. Moreover, we revealed increased phosphorylation of heat shock protein 27 (Hsp27) upon serum treatment, which was diminished by p38-MAPK-inhibition. In summary, we demonstrate human blood serum as a strong inducer of adult human cardiac stem cell migration dependent on p38-MAPK/Hsp27-signalling. Our findings further emphasize the great potential of microfluidic cultivation devices for assessing spatio-temporal migration dynamics of adult human stem cells on a single-cell level.

## 1. Introduction

Adult human stem cells (ASCs) can be found in various tissues of the human body where they remain as quiescent cells in their respective niches and upon activation contribute to tissue renewal and regeneration [1]. To reach the exact locations of damaged tissue, ASCs often exhibit a migratory behavior, referred to as homing [2]. The underlying mechanisms of this process are well described, and migration requires a deformation of the cell shape, which is achieved by reorganization of the actin cytoskeleton. Here, a highly orchestrated cascade of actin polymerization drives the formation of protrusions and the adhesion to a substrate or extracellular matrix (ECM) at the leading zone of cell movement [3].

In recent years, various stimuli have been identified that induce or inhibit migratory behavior in diverse cell populations. As well as mechanical factors like shear stress, matrix stiffness or mechanical strain [4,5,6,7], a range of chemokines, cytokines and growth factors is involved in the regulation of stem cell migration behavior [8]. 

The application of human blood plasma and serum is a therapeutic approach chosen in cases of impaired wound healing [9], coagulopathy [10], and liver cirrhosis [11]. Moreover, the use of convalescent plasma is currently under discussion as a treatment option in the COVID-19 pandemic [12,13]. In vitro cultivation of adult human stem cells with human blood plasma or serum has demonstrated increased proliferation and viability [14,15,16,17]. Mishima and coworkers showed increased migration of human articular chondrocytes and mesenchymal stem cells (MSCs) in response to 5, 10 and 20% fetal bovine serum (FBS) [18]. Human keratinocytes were demonstrated to respond to human serum treatment with increased migration dependent on activation of p38 mitogen-activated protein kinase (p38-MAPK) [19]. In contrast, human serum inhibited the migration of human fetal skin fibroblasts and fetal lung fibroblasts [20,21]. These data demonstrate that the effect of human serum on cell migration strongly depends on the cell type. In this context, the influence of human blood serum on the migration behavior of adult human stem cells is only poorly described. In the present study, we assessed potential effects of blood serum on migration of adult human cardiac stem cells.

Adult human cardiac stem cells (hCSCs) were first described in 2007 as a multipotent population residing in the adult human myocardium [22]. However, their in vivo contribution to cardiac regeneration remains unclear and is highly discussed [23]. Therefore, investigations that describe the migratory behavior of adult human cardiac stem cells could make important contributions to the current knowledge of cardiac stem cell behavior. Very recently, we identified a human cardiac stem cell population derived from the left atrial appendage of the adult heart [17,24]. These cells express common cardiac stem cell markers like Sca1 and also markers that are associated with the neural crest. Likewise, their differentiation potential extends the cardiogenic lineage to a differentiation capacity into the adipogenic, neurogenic and osteogenic derivates. We therefore concluded these cells to be a novel neural crest-derived cell population isolated from the adult heart. In order to develop new stem cell-based therapeutic strategies addressing human heart protection and regeneration, more research is necessary to investigate the regenerative capacities of hCSCs. To our knowledge, little is known about homing and migration of adult cardiac stem cells. However, we showed that human blood plasma and serum have beneficial effects on hCSC viability in terms of inducing cell proliferation and protection from senescence. Moreover, we showed the p38-MAPK pathway to be highly important in this process [17]. The p38-MAPK pathway has been described as an important player in the regulation of migration in various cell types [25,26,27,28,29]. Recently, Dubon and colleagues showed that p38-MAPK mediated the migrative response of murine MSC-like ST2-cells to TGF-β1 [29]. P38-MAPK was also shown to be crucial for human umbilical cord blood-derived MSC migration [26]. Within this study, we aimed particularly to investigate the effects of human blood serum on hCSC migration and a potential role of p38-MAPK within this process. Since cell migration depends on the reorganization of the actin cytoskeleton, chaperones such as the heat shock protein 27 (Hsp27) play a central role within this process. Hsp27 has been shown to be involved in F-actin assembly and migrative processes of vascular smooth muscle cells [30,31]. However, other studies demonstrated apoptosis of lung squamous cell carcinoma cells upon phosphorylation of Hsp27 [32], which indicates a potential cell type-specific function of Hsp27.

On a technical level, experiments assessing cell migration have been mainly carried out to date as scratch wound assays or by compartmentalization of the cultivation wells with transwell inserts [8]. These assays are often limited to an end-point determination of entire populations concerning migration distance or direction and rarely allow any insights into single-cell behavior. Further, standard cell culture dishes or flasks only operate in batch-mode, and cells are exposed to changing environmental conditions during the experiment. Facing these challenges, a range of microfluidic approaches have been developed [33,34,35]. Because of their ease of use, most prominent is the utilization of polydimethylsiloxane (PDMS)-based devices, which additionally facilitate the application of microscopic live cell imaging because of their optical transparency [36,37]. Decreasing the experimental scale from the milliliter to nanoliter range furthermore reduces the volume of a sample needed and reagent consumption, making microfluidic approaches especially beneficial in case of expensive reagents or rare samples such as patient-specific cells [38]. Since most microfluidic devices are operated under steady fluid flow, they come with a high level of environmental control, resulting in constant assay conditions, or even allow dynamic changes between various conditions [39].

Different approaches aiming at scaling down conventional scratch wound or compartmentalization assays to the microscale can be found in the literature. Similar to ordinary transwell assays, cells can be cultivated with artificial gaps in a confluent monolayer by means of removable PDMS barriers [40] or microstencils [41]. Extracting these micrometer-scale exclusion structures leads to a cell-free zone, and migration of adjacent cells can be analyzed. As an analogy to scratch wound assays, linear microchannels have been developed which exhibit multiple inlets, leading to independent fluid zones due to laminar flow characteristics in microfluidic devices [42,43,44,45]. In a first step, cells are grown confluently throughout the whole microchannel by offering standard cultivation conditions. In a second step, trypsin is flushed through a portion of the microchannel resulting in cell detachment in this zone. In a final step, the cell-free zone is rinsed with cultivation medium or treatment solution again and cell migration into the cell-free zone can be analyzed. Alternatively, microfluidic setups with defined migration channels, where contrary to microchannels, single cells are applied to study cellular migration, have been reported [7,46,47]. These setups are particularly suitable for the analysis of chemoattractants, as gradient formation in microchannels is reliably realizable.

In terms of migration studies, only few microfluidic devices show strict separation of growth and supply zones due to chamber-based designs. Yet compartmentalization restricts single-cell growth and motility to selected positions on the microfluidic device and thereby not only allows a high temporal, but also spatial resolution of cellular behavior [48]. Due to single-cell loading of different chambers, cells can be analyzed in an isolated way and thus experimental outputs represent single-cell reactions instead of average population behavior.

Within this study, single-cell behavior of primary adult human cardiac stem cells (hCSCs) inside a previously introduced microfluidic single-cell cultivation device [48] was tracked and analyzed for up to 48 h under controlled environmental conditions. Here, we provide an overview of different cell morphologies during migration, cell division and cell death. We further traced the migration distance and speed of hCSCs, dependent on human blood serum, for up to 48 h. After application of human blood serum, the distance, as well as the migration speed, of hCSCs was significantly increased compared to untreated cells. Moreover, we demonstrated a p38-dependent activation of migratory behavior upon serum treatment. Immunocytochemical staining further showed a p38-dependent phosphorylation of Hsp27 in hCSCs treated with human serum. The presented method offers the possibility to directly track the response of primary adult human stem cells in terms of their spatio-temporal migration dynamics and morphology upon treatment with different stimuli.

## 2. Materials and Methods

### 2.1. Isolation and Cultivation of Human Cardiac Stem Cells

**Figure 1:**
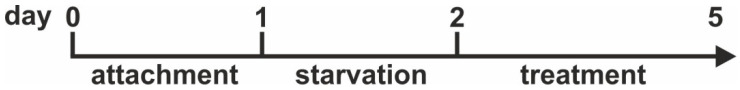
Consecutive cultivation conditions of hCSCs in the microfluidic cultivation device.

Adult human cardiac stem cells (hCSCs) were derived from left atrial appendages that were isolated during routine heart surgery according to local and international guidelines (declaration of Helsinki) after informed and written consent. Isolation and further experimental procedures were ethically approved by the ethics commission of the Ruhr-University Bochum (Faculty of Medicine, located in Bad Oeynhausen) (approval reference number eP-2016-148). Isolation and cultivation of hCSCs were carried out as described before [17,24]. After precultivation and expansion of hCSCs in a T25 cell culture flask (Sarstedt AG and Co., Nürmbrecht, Germany), cells were detached using Trypsin-EDTA (Sigma Aldrich, St.-Louis, MO, USA) and a suspension of approx. 10^6^ cells/mL was prepared for loading a microfluidic cultivation device. After successful seeding of hCSCs in the cultivation chambers, cells were allowed to attach to the surface for 24 h in hCSC-medium containing DMEM/F12 (Sigma Aldrich, St. Louis, MO, USA) supplemented with 10% fetal calf serum (VWR, Radnor, PA, USA), 10 mg/mL penicillin/streptomycin (Sigma Aldrich), 200 mM L-glutamine (Sigma-Aldrich), 5 ng/mL basic fibroblast growth factor (bFGF) (Peprotech, Hamburg, Germany) and 10 ng/mL epidermal growth factor (EGF) (Peprotech) (Figure 1). In the starvation period, the medium was switched to starvation-medium consisting of DMEM/F12 (Sigma Aldrich) supplemented with 10 mg/mL penicillin/streptomycin (Sigma Aldrich), 200 mM L-glutamine (Sigma-Aldrich), 5 ng/mL basic fibroblast growth factor (bFGF) (Peprotech, Hamburg, Germany) and 10 ng/mL epidermal growth factor (EGF) (Peprotech) for 24 h. In the treatment phase, the starvation medium was supplemented with either 10% human blood serum or 10% human serum and 50 μM p38-MAPK-inhibitor SB239063 (Medchemexpress, Sollentuna, Sweden) in accordance with our previous study [17]. Blood plasma was collected from routine blood donation service from healthy individual donors. For the isolation of serum from fresh frozen plasma (FFP), 20% CaCl_2_ was added in a ratio of 1:50 and incubated at 4 °C overnight. After centrifugation at 1920 RCF for 20 min, blood serum was harvested from the supernatant.

### 2.2. Microfluidics

The applied microfluidic PDMS-glass cultivation device was fabricated in a multiple-step procedure as described previously [48]. Using photolithographic techniques, a silicon wafer was fabricated in clean room facilities. In a following soft lithography step, PDMS base and curing agent (SYLGARD 184 Silicone Elastomer, Dow Corning Corporation, Midland, MI, USA) were mixed in a 10:1 ratio and PDMS chips were molded from the wafer. After an intermediate cleaning step, the PDMS chip and a glass substrate were surface-activated via oxygen plasma and covalently bonded to each other. Microfluidic cultivation of single hCSCs was performed on an automated inverted microscope (Nikon Eclipse Ti2, Nikon Instruments, Düsseldorf, Germany) for multiple days. To assure steady cultivation conditions, a microscope incubator system (Cage incubator, OKO Touch, Okolab S.R.L., Ottaviano, Italy) and additional CO_2_ incubation chamber (H201-K-FRAME GS35-M, Okolab S.R.L.) were applied, guaranteeing a constant cultivation temperature of 37 °C and an atmosphere of 5% CO_2_. Time-lapse images of relevant positions were taken periodically to monitor cellular morphology and migration behavior via phase-contrast microscopy, using a 40× objective (NIS Elements AR 5.20.01 Software, Nikon Instruments, Düsseldorf, Germany).

By manually flushing the microfluidic cultivation device with cell suspension, hCSCs were seeded into the respective cultivation chambers until loading was sufficient. Subsequently, single-use syringes containing cultivation medium were connected via PTFE tubing to the microfluidic chip, and medium supply with a constant flow rate of 2 μL/min was established by the use of low-pressure syringe pumps (neMESYS, CETONI, Korbussen, Germany).

### 2.3. Immunocytochemistry

For immunocytochemistry, hCSCs were seeded in ibidi-μ-slides (ibidi GmbH, Gräfelfing, Germany) and treated as described above (Figure 1; Section 2.1). Biological replicates were performed of two individual serum donors. Cells were fixed in 4% paraformaldehyde (PFA), washed with phosphate-buffered saline (PBS) (Sigma Aldrich) and permeabilized in PBS with 0.02% Triton X-100 (Applichem, Darmstadt, Germany) supplemented with 5% goat serum for 30 min. The primary antibodies were diluted in PBS (mouse anti-Nestin 1:200 (Millipore, Burlington, MA, USA), rabbit anti-PhosphoHsp27 (Ser82) (Cell Signaling Technology, Danvers, MA, USA)) and applied for 1 h at room temperature (RT). After three washing steps, secondary fluorochrome-conjugated antibodies (Alexa 488 anti-mouse or Alexa 555 anti-rabbit, Invitrogen, Life Technologies GmbH, Carlsbad, CA, USA) were applied for 1 h at RT with a dilution ratio of 1:300. Nuclear staining was realized by incubation with 4,6-diamidin-2-phenylindol (DAPI) (1 μg/mL, Applichem) in PBS for 15 min at RT. Finally, the samples were mounted with Mowiol (self-made). Imaging was performed using a confocal laser scanning microscope (CLSM 780, Carl Zeiss, Oberkochen, Germany). Five images were taken of each treatment condition and serum-donor for following data analysis.

### 2.4. Data Analysis

Following microfluidic single-cell cultivation, microscope images were exported as 8-bit TIFF images and processed with ImageJ/Fiji software to create videos [49]. The manual tracking plugin was used to visualize the migration paths of the cells and the measure function was used to analyze the mean fluorescence intensity in the CLSM images. The corresponding data were statistically analyzed with Prism software (GraphPad Software, San Diego, CA, USA).

## 3. Results

### 3.1. Successful Cultivation of Human Cardiac Stem Cells in a Microfluidic Cultivation Device

To access the ability of hCSCs to survive and proliferate in a microfluidic cultivation device, the established MaSC platform [48] was adapted to the cultivation and analysis of single adult cardiac stem cells. 

**Figure 2:**
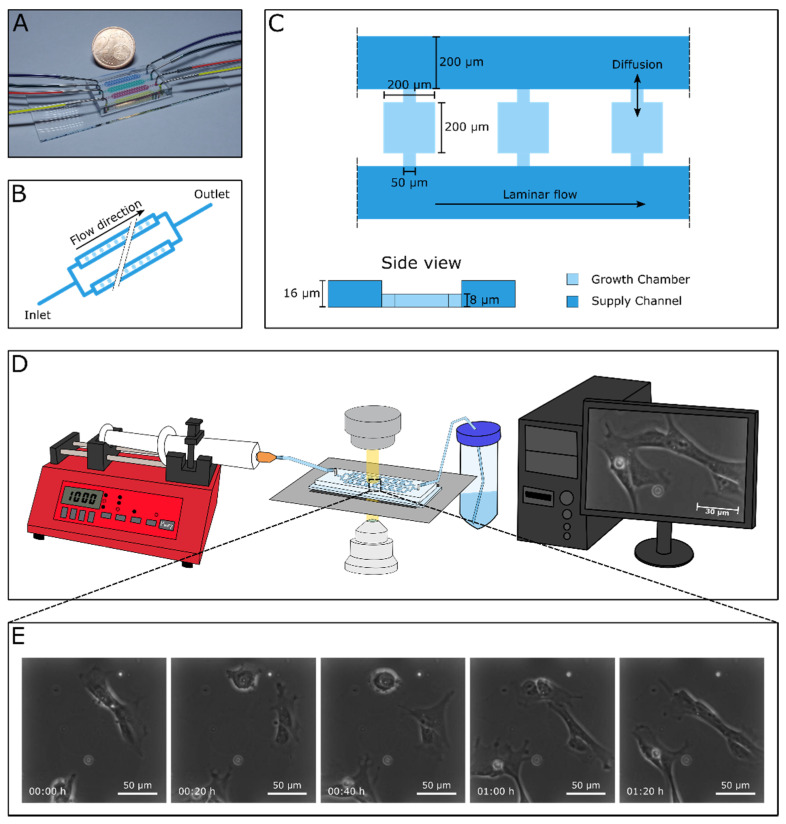
Chip design and working principle of the experimental setting. (**A**) Microfluidic cultivation device consisting of four cultivation arrays for the analysis of hCSCs. (**B**) Schematic figure of one cultivation array. Each array includes four parallel supply channels with 30 cultivation chambers between two of them, resulting in a total number of 60 chambers per array. (**C**) Three exemplary cultivation chambers with an area of 200 × 200 μm^2^ and a height of 8 μm. The adjacent supply channels with a width of 200 μm are twice as high. Mass transport from supply channel into the cultivation chambers is almost exclusively diffusive. (**D**) Experimental setup showing the syringe pump for steady medium supply, the microfluidic cultivation device mounted onto an inverted microscope, and the computer-assisted automated live cell imaging. (**E**) Time-lapse image sequence illustrating cellular behavior of single hCSCs.

The applied device consisted of four parallel-arranged independent cultivation arrays (Figure 2A) which simultaneously enabled four different experiments. For seeding hCSCs into the device, we prepared a solution of approx. 10^6^ cells/mL in hCSC-medium. Cell suspension was flushed into each array through the outlet by means of a 1 mL single-use syringe and cells were seeded into the respective cultivation chambers by manually moving the suspension back and forth through the adjacent supply channels (Figure 2B). Since there was no flow inside the chambers, single cells entered the chambers randomly. Following successful loading, pumping periphery was connected to the device’s inlets and the flow-through was collected in a waste tube, which then was connected to the arrays’ outlets. Due to a constant perfusion of the microfluidic device with a flow rate of 2 μL/min, seeded cells were continuously supplied with fresh hCSC-medium guaranteeing consistent cultivation conditions. Mass transport inside the chambers with a dimension of 200 × 200 μm^2^ (200 × 350 μm^2^) and a height of 8 μm was almost exclusively diffusive. Flow inside the chamber was additionally restricted by the difference in height between the cultivation chambers and the supply channel (Figure 2C). Therefore, hCSC cells were not exposed to any shear stress inside the cultivation chambers. Positions of cell-containing chambers were marked in the software and the microscope was programmed to record images of each position in a preset interval (Figure 2D). We detected a high number of cells attached to the PDMS-surface, which were migrating and proliferating (Figure 2E, Supplementary Videos S1 and S2).

### 3.2. Migrating hCSCs Exhibit Diverse Morphologies and Migration Patterns

During cultivation in the microfluidic device and accompanying image acquisition of single cells, we were able to observe various morphologies and migration behavior such as a mesenchymal-like shape (Supplemental Figure S1A) or amoeboid-like migration along the walls of the cultivation chambers (Supplemental Figure S1B). Further, some migrating cells also exhibited a flattened morphology with pseudopodia or lamellipodia in the leading zone (Supplemental Figure S1C). These different morphologies were presented alternately by the same individual cells, making the underlying mechanisms and signaling pathways highly interesting for future studies. We further detected temporary cell-cell-contacts between neighboring cells by the formation of stretches reaching out to the other cells, and the subsequent withdrawal of these stretches (Supplemental Figure S1D, Supplementary Video S3). Especially in the attachment phase, and for cells exposed to serum in the treatment phase, we could detect events of cytokinesis where a cell first exhibited a sphere-like morphology followed by the formation of a cleavage furrow and the stretching of the two daughter cells to a flattened shape with leading zone lamellipodia migrating away from each other (Supplemental Figure S1E, Supplementary Video S4). Events of cell death were visible in each treatment group but especially in the group of cells treated with serum-free medium. Dying cells formed a rounded shape with disheveled margins resulting in lysis or release of the cytoplasm (Supplemental Figure S1F, Supplementary Video S5). Although we could not analyze these morphologies and events in a quantitative manner, we provide exemplary images and schemes of these different events and morphologies to present for the first time an overview of the different morphologies of adult human cardiac stem cells during *in vitro* cultivation.

### 3.3. Human Blood Serum Enhances the Migration Distance and Speed of hCSCs

**Figure 3:**
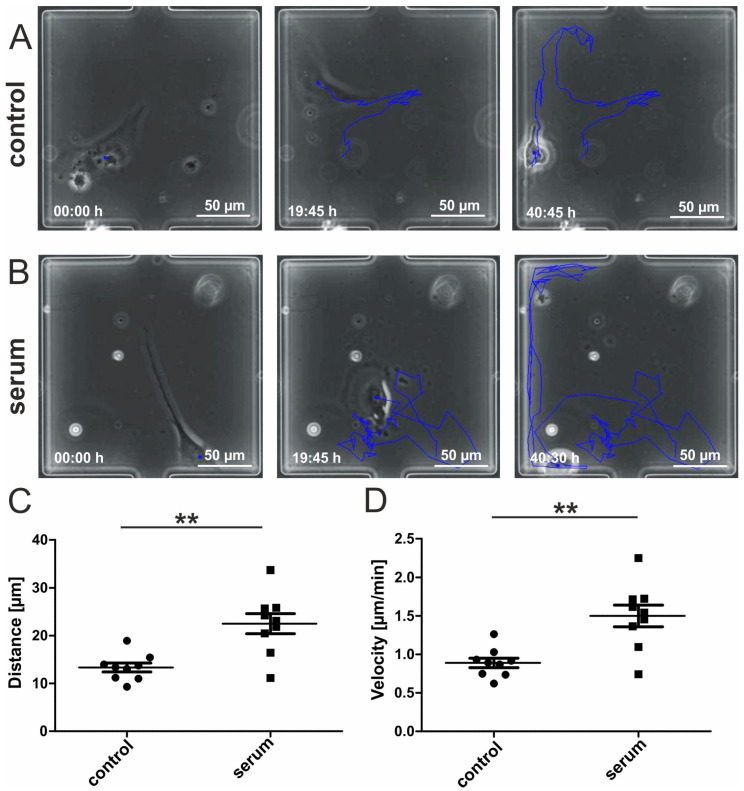
Human blood serum enhances migration behavior of hCSCs. (**A**) Exemplary images of hCSCs cultivated without serum directly after medium switch, after 19:45 h and after 40:45 h. The blue line indicates the migration path. (**B**) Exemplary images of hCSCs cultivated with human blood serum directly after medium switch, after 19:45 h and after 40:30 h. The blue line indicates the migration path. (**C**) Migration distance of hCSCs is significantly increased by the application of human serum. (**D**) Migration velocity of hCSCs is significantly increased by the application of human serum. Data points represent migration dynamics of single hCSCs observed in individual cultivation chambers. Measurements were performed with hCSCs from one donor and blood serum from one donor. Mann-Whitney one-tailed, ** *p* < 0.005 was considered significant.

We next applied a starvation phase of 24 h by switching the syringe connected to the arrays’ inlets from FCS-containing hCSC-medium to serum-free medium. Here, hCSCs remained viable and attached to the surface. In the following treatment period, the syringes with the medium of one array were switched to medium containing 10% human blood serum. In vitro cultivation of adult human stem cells with human blood plasma or serum has demonstrated increased proliferation and viability [14,15,16,17]. To investigate a potential effect of human serum on hCSC migration, we tracked the covered path of single cells that were cultivated under exposure to blood serum or to control medium in our microfluidic device. We captured images of marked positions every 15 min and analyzed these data using the manual tracking plugin of ImageJ [49]. HCSCs showed migration behavior in the cultivation chambers under each cultivation condition independent of the cultivation time, although on average the migration activity of serum treated hCSCs was highly increased (Figure 3A,B) (Supplemental Figure S2, Supplementary Video S6). The resulting data allowed us to statistically compare the migration distance and velocity of the two treatment groups. The covered track of serum-treated hCSCs was significantly longer than of hCSCs treated with serum-free medium (Figure 3C). Further, serum treatment significantly increased the velocity of migrating hCSCs compared to untreated hCSCs (Figure 3D). These results encouraged us to functionally analyze putative underlying pathways involved in the blood serum-mediated increase of hCSC-migration. 

### 3.4. Inhibition of p38-MAPK Leads to Decreased Migration of Blood-Serum Stimulated hCSCs

**Figure 4:**
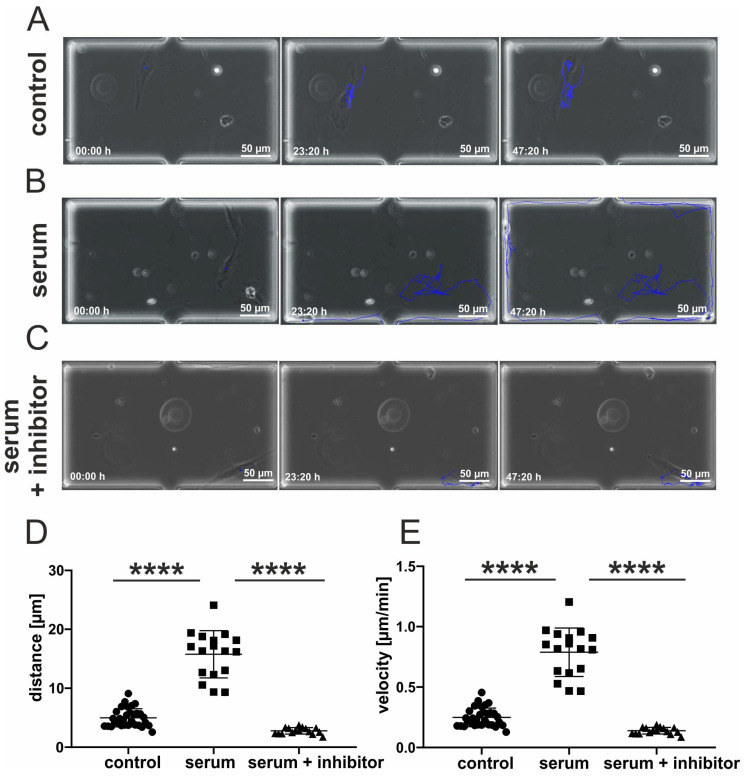
Increased migration behavior of hCSCs after application of human blood serum is dependent on p38-MAPK signaling. (**A**) Exemplary images of hCSCs cultivated without serum directly after medium switch, after 23:20 h and after 47:20 h. The blue line indicates the migration path. (**B**) Exemplary images of hCSCs cultivated with human blood serum directly after medium switch, after 23:20 h and after 47:20 h. The blue line indicates the migration path. (**C**) Exemplary images of hCSCs cultivated with human blood serum and p38-MAPK-inhibitor SB239063 directly after medium switch, after 23:20 h and after 47:20 h. The blue line indicates the migration path. (**D**) Migration distance of hCSCs is significantly increased by the application of human serum. This effect is reversed by the inhibition of p38. (**E**) Migration velocity of hCSCs is significantly increased by the application of human serum. This effect is reversed by inhibition of p38. Data points represent migration dynamics of single hCSCs observed in individual cultivation chambers. Measurements were performed with hCSCs from one donor and blood serum from one donor. Mann-Whitney one-tailed, **** *p* < 0.0001 was considered significant.

We recently described a blood serum-mediated effect on hCSC proliferation and senescence which is party mediated by p38-signaling [17]. These data motivated us to test a possible influence of p38-MAPK on the blood serum mediated migration of hCSCs. We therefore performed a microfluidic experiment where p38-MAPK-inhibitor SB239063 was applied to hCSCs along with human blood serum. Cells cultivated in serum-free medium exhibited a moderate migration track (Figure 4A), while the application of human serum led to increased migration (Figure 4B). Interestingly, cells cultivated in the presence of human blood serum and the p38-MAPK-inhibitor showed almost no migration accompanied by a mesenchymal-like morphology (Figure 4C, Supplementary Video S7). Quantitative assessment of the covered tracks showed a significant increase in the migrated distance of serum-treated hCSCs compared to untreated cells. This effect was reversed after application of the p38-MAPK-inhibitor SB239063, which resulted in a significantly decreased migration distance of hCSCs compared to their serum-treated counterparts (Figure 4D). We likewise observed the strong increase in migration velocity of serum-treated hCSCs (compared to untreated cells) to be significantly reduced by additional inhibition of p38-MAPK (Figure 4E).

### 3.5. Serum-Induced Migration of hCSCs Is Regulated via p38-MAPK and Hsp27-Phosphorylation

To further investigate a potential mechanism by which p38-MAPK controls the serum-induced migration of hCSCs, we examined the phosphorylation of heat shock protein 27 (Hsp27), a common target of p38-MAPK in migration in various cell types [30]. However, the migrative activity of cardiac stem cells and its underlying pathways has not yet been described in detail. Here, we performed immunocytochemical staining of phosphorylated Hsp27 (phospho-Hsp27) to visualize the activated form of Hsp27. 

**Figure 5:**
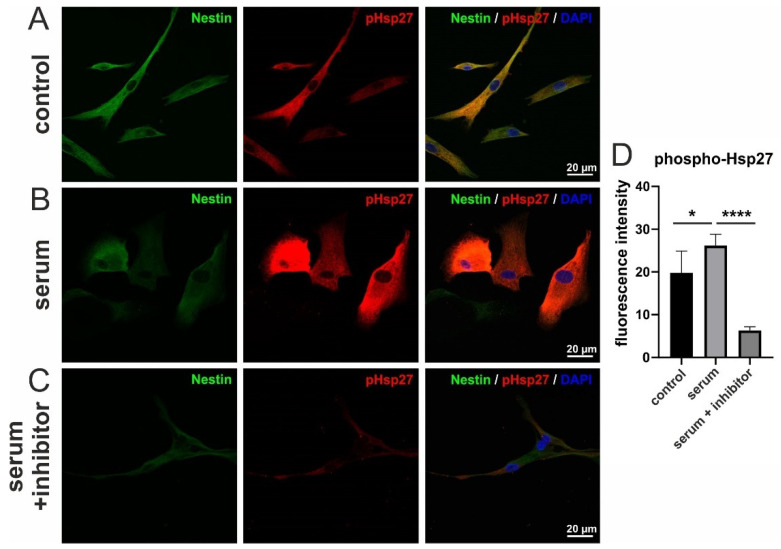
Immunocytochemistry of Nestin and phospho-Hsp27 in hCSCs treated with human serum and p38-MAPK-inhibitor. (**A**) Untreated cells exhibit a basal level of Hsp27 phosphorylation. (**B**) Phosphorylation of Hsp27 in serum-treated cells is strongly increased. (**C**) Hsp27-phosphorylation is inhibited after the application of the p38-MAPK inhibitor SB239063. (**D**) Quantification of fluorescence intensity across all replicates. Measurements were performed with hCSCs from one donor and blood serum from two donors. Mann-Whitney one-tailed, * *p* < 0.05; **** *p* < 0.0001 was considered significant.

A basal level of Hsp27-phosphorylation was observable in untreated cells (Figure 5A(middle panel)). Interestingly, hCSCs treated with human serum showed a significantly higher degree of Hsp27-phosphorylation (Figure 5B(middle panel)), indicating Hsp27 as a target of human serum-induced signaling within hCSCs. To test whether the serum-induced phosphorylation of Hsp27 was dependent on p38-MAPK, we additionally applied the p38-MAPK-inhibitor SB239063 together with human serum to hCSCs. Importantly, p38-MAPK inhibition significantly decreased the level of Hsp27-phosphorylation to an amount even lower than in untreated cells (Figure 5C(middle panel),D, Supplemental Figure S3). Interestingly, the level of the intermediate filament and NCSC-marker Nestin seems to be unaffected after serum treatment in comparison to untreated cells but slightly reduced after the simultaneous application of human serum and p38-MAPK inhibitor (Figure 5A–C).

In summary, these data indicate a potential regulation of hCSC migration upon serum-treatment via p38-MAPK and Hsp27-phosphorylation.

## 4. Discussion

The present study describes the beneficial effects of human blood serum on human cardiac stem cell migration by implementing a microfluidic device established for single-cell cultivation in contrast to customarily applied miniaturized scratch wound or transwell assays. We particularly observed a significant increase of hCSC migration distance and speed upon exposure to human blood serum. On a mechanistic level, we demonstrated the beneficial effects of human serum to be dependent on Hsp27 phosphorylation, which is reversable by inhibition of p38-MAPK signaling.

During cultivation in the microfluidic device, we observed a range of different morphologies such as mesenchymal or amoeboid-like cell shapes. HCSCs also presented fan-like lamellipodia at the leading edges. These observations are in line with a range of studies describing the mechanisms of actin polymerization leading to protrusions and integrin attachment to the cultivation surface [3]. Accordingly, we recently detected an upregulation of genes associated to the KEGG pathways ‘focal adhesion’ and ‘ECM-receptor interaction’ in untreated hCSCs compared to non-adherent human hematopoietic stem cells [24]. Within this study, single hCSCs demonstrated a high plasticity by switching between the different morphologies during cultivation. This mode switching was also observed in human fibroblasts and thought to be associated with the speed or directionality of migrating cells and may be dependent on external cues such as the composition of the extracellular matrix (ECM) or soluble signaling factors [50,51,52]. However, in absence of changing external cues, internal cues were thought to be responsible for migration mode switching [52]. Our established microfluidic platform further allowed us to observe distinct events like cell division and cell death. Dying cells showed disheveled margins with blebs around the surface. These blebs are commonly observed in apoptotic cells before tying off apoptotic bodies as a result of ROCK1 cleavage and actomyosin contraction leading to delamination of the plasma membrane from the cytoskeleton membrane [34,53,54].

Next to a detailed description of the diverse morphologies of hCSCs during cultivation, we investigated the migration behavior dependent on human blood serum. Recently, we described increased proliferation and decreased senescence of human serum-treated hCSCs [17]. Here, we extended these findings from proliferation and senescence studies by the investigation of cellular migration as another important parameter of stem cell functionality. For the first time, we could detect a significantly increased migration distance and velocity of hCSCs after application of human serum. In accordance with our data, a serum-dependent migration was also reported in human articular chondrocytes and MSCs cultivated in 5, 10 and 20% fetal bovine serum [18]. In contrast, migration of human fetal skin fibroblasts and fetal lung fibroblasts was inhibited after application of human blood serum [20,21]. These contradicting data strongly indicate a cell type-dependent response to human serum treatment. In this regard, in the present study we demonstrated for the first time the beneficial effects of human serum on the migration of adult human cardiac stem cells in vitro.

**Figure 6:**
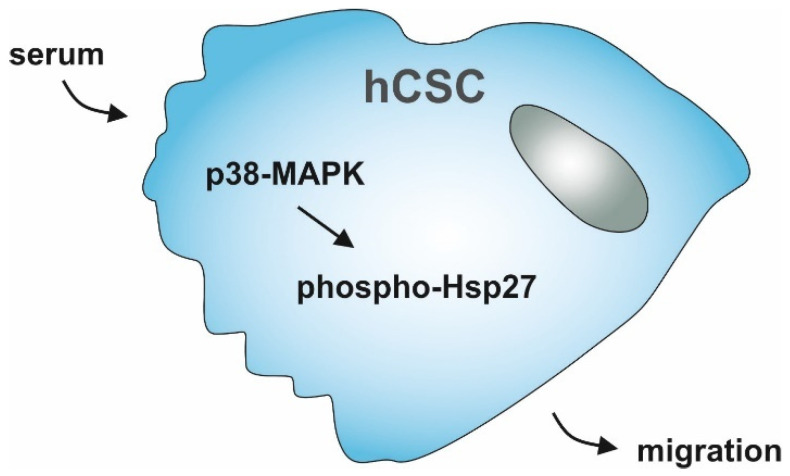
Schematic summary of hCSC-migration upon serum-treatment regulated by p38-MAPK and Hsp27.

To gain functional insights into the increase in cell migration distance and velocity in human serum treated hCSCs, we applied the p38-MAPK inhibitor SB239063 together with serum in our microfluidic cultivation device. Here, p38-inhibition led to a reversal of serum-induced migration, strongly indicating a participation of p38-signaling in this process. In accordance with our observations, the p38-MAPK pathway has been shown to be important for migration in a range of other cell types. For instance, Dubon and coworkers showed increased migration of murine MSC-like ST2-cells as response to TGF-β1-treatment mediated by p38-MAPK [29]. Further, human umbilical cord blood-derived MSCs migration was reported to be directed by p38-MAPK [26]. Hamanoue and colleagues described a p38-dependent migration of mouse neural stem cells [27]. Other groups reported p38-activation in highly proliferating and migrating MDA-MB-231 breast cancer cells [28], or p38-induced alterations in actin architecture and corresponding migration of human umbilical vein endothelial cells (HUVECs) [25]. Interestingly, our findings may also indicate Nestin protein amounts to be affected by p38-MAPK-inhibitor treatment, although we previously showed no differential gene expression levels of Nestin mRNA between hCSCs treated with serum compared to untreated hCSCs [17]. With regard to the pivotal role of p38-MAPK in migration behavior of hCSCs observed here, we further showed significantly increased phosphorylation of the p38-MAPK target Hsp27 in hCSCs after treatment with human serum. The chaperone Hsp27 is a common target of p38-MAPK signaling, along with other cellular processes being involved in F-actin assembly and thus in migrative processes [30,55]. Huang and colleagues indicated Hsp27 might to be involved in the regulation of actin reorganization and thus migration of vascular smooth muscle cells [31]. The increased phosphorylation of Hsp27 in serum-treated hCSCs observed in the present study is in line with these findings. In addition, simultaneous application of the p38-MAPK-inhibitor SB239063 and human serum significantly decreased the level of Hsp27 phosphorylation. These results clearly demonstrate the serum-induced migration of hCSCs to be regulated via the p38-MAPK and Hsp27 axis (Figure 6).

Phosphorylation of Hsp27 was further shown to be necessary for F-actin formation and stabilization of focal adhesions in vascular smooth muscle cells [30]. Interestingly, a global gene expression analysis of blood serum-treated hCSCs showed the KEGG pathway ‘focal adhesion’ being significantly enriched along with ‘MAPK signaling pathway’. Further, the GO-terms ‘p38 MAPK-pathway’ and ‘Integrin signaling pathway’ were found to be enriched in serum treated hCSCs compared to untreated hCSCs [17]. In contrast, a recent study showed p38-MAPK and phosphorylated Hsp27 to mediate apoptosis of lung squamous cell carcinoma cells [32], suggesting a potential cell type-specific function of p38-MAPK and Hsp27. These findings also underline the necessity to investigate the molecular dynamics of migration in a cell type-specific manner. For adult human cardiac stem cells, our data reveal a central role of p38-MAPK signaling and Hsp27 phosphorylation in their serum-dependent migration and may enable new potential therapeutic approaches to enhance cardiac regeneration in heart failure patients. 

In comparison to the most prominently employed in vitro cell migration assays, namely scratch wound assays or transwell inserts [8], as well as their microfluidic versions [41,44], the microfluidic approach presented in this study comes with multiple benefits. Common end-point determination of migrated cells, leaving the meantime dynamics in migration direction or morphology undetected, are replaced by highly time-resolved analysis, due to the application of live cell imaging [39]. Especially in the case of cellular response to certain stimuli such as human serum, the presented method provided valuable insights into single-cell decisions which would have stayed masked using typical average end-point measurements. In contrast to frequently applied microfluidic channels for cell migration investigation, where no compartmentalization is applied, and thus only population behavior can be analyzed [42], our chamber-based design allows the analysis of single-cell dynamics in spatially separated compartments. Another significant advantage of the chamber-based design are the uniform treatment conditions throughout the whole device in comparison to the analysis of cellular behavior at laminar boundary layers, which can be seen with approaches relying on laminar flow-based reaction zones [45]. In combination with steady perfusion of the presented microfluidic cultivation device, full environmental control was guaranteed and made single-cell analysis under changing cultivation conditions possible for several days. Former microfluidic devices were limited to experimental durations of max. 12 h by either their stopped-flow operated chamber-based design [56] or their limited migration channel length [7].

Cell-to-cell heterogeneity described in primary adult stem cell populations [57,58,59,60] is increasingly noted as a crucial parameter determining their behavior and becomes approachable due to the long-term cultivation mode of our microfluidic device and its high spatio-temporal resolution of cellular responses. In this regard, our microfluidic approach may also serve for determining the heterogeneous behavior of other neural crest-derived adult stem cell populations [60], mesenchymal stem cells, or even cancer stem cells. Since cancer stem cells reveal a broad heterogeneity [61] and highly migratory behavior, the applied microfluidic method holds great promise to assess their heterogeneous behavior on a single-cell level. Furthermore, not only cell migration but also the tracking of cell-to-cell interaction or differentiation might be addressable due to the chamber-based design of our microfluidic cultivation device.

## 5. Conclusions

In summary, this study demonstrated a beneficial effect of human blood serum on the migration behavior of an adult human cardiac stem cell population in a p38-MAPK/phospho-Hsp27-dependent manner. We further provided a microfluidic-based cultivation method facilitating the measurement of primary human stem cell migration. A well as an overview of different morphologies of hCSCs during *in vitro* migration, we showed that human serum significantly enhances hCSC migration distance and speed and increased phosphorylation of Hsp27. Application of a specific inhibitor of p38-MAPK completely reversed this effect, strongly suggesting that the activation of hCSC migration by human serum is mediated by p38-signaling and subsequent Hsp27-phosphorylation. The presented method offers the possibility to directly track the response of primary adult human stem cells in terms of their spatio-temporal migration dynamics and morphology upon treatment with different stimuli. In this regard, our microfluidic approach may be a valuable tool for future applications assessing the migration dynamics of other adult stem cell populations such as mesenchymal stem cells or even cancer stem cells. As we show, for the first time, increased migration dynamics of human cardiac stem cells by human blood serum in a p38-MAPK-dependent manner, our study also builds the basis to further investigate molecular networks underlying cardiac stem cell migration.

## Data Availability

The data presented in this study are available on request from the corresponding author. The data are not publicly available due to the size of the respective image data (multiple gigabytes).

## Supplementary Materials

The following are available online at https://www.mdpi.com/article/10.3390/biology10080708/s1. Figure S1: Diverse morphologies and migration modes of hCSCs, Figure S2: Migration velocity of two exemplary individual cells over time, Figure S3: Immunocytochemistry of hCSCs treated with p38-MAPK-inhibitor only, Video S1: Attachment of hCSCs after loading onto the microfluidic chip, Video S2: Successful cultivation of hCSCs in a microfluidic chip, Video S3: Temporary cell-cell-contacts between adjacent cells, Video S4: Cytokinesis, Video S5: Cell death. Video S6: Human serum increases hCSC migration behavior, Video S7: p38-MAPK-inhibitor reverses blood serum-mediated hCSC-migration.
