# Supplementary figures and images for: Human Blood Serum Induces p38-MAPK- and Hsp27-Dependent Migration Dynamics of Adult Human Cardiac Stem Cells: Single-Cell Analysis via a Microfluidic-Based Cultivation Platform
Source: Biology (Basel). 2021 Jul 24;10(8):708. doi: 10.3390/biology10080708 (PMC8389316; doi:10.3390/biology10080708)

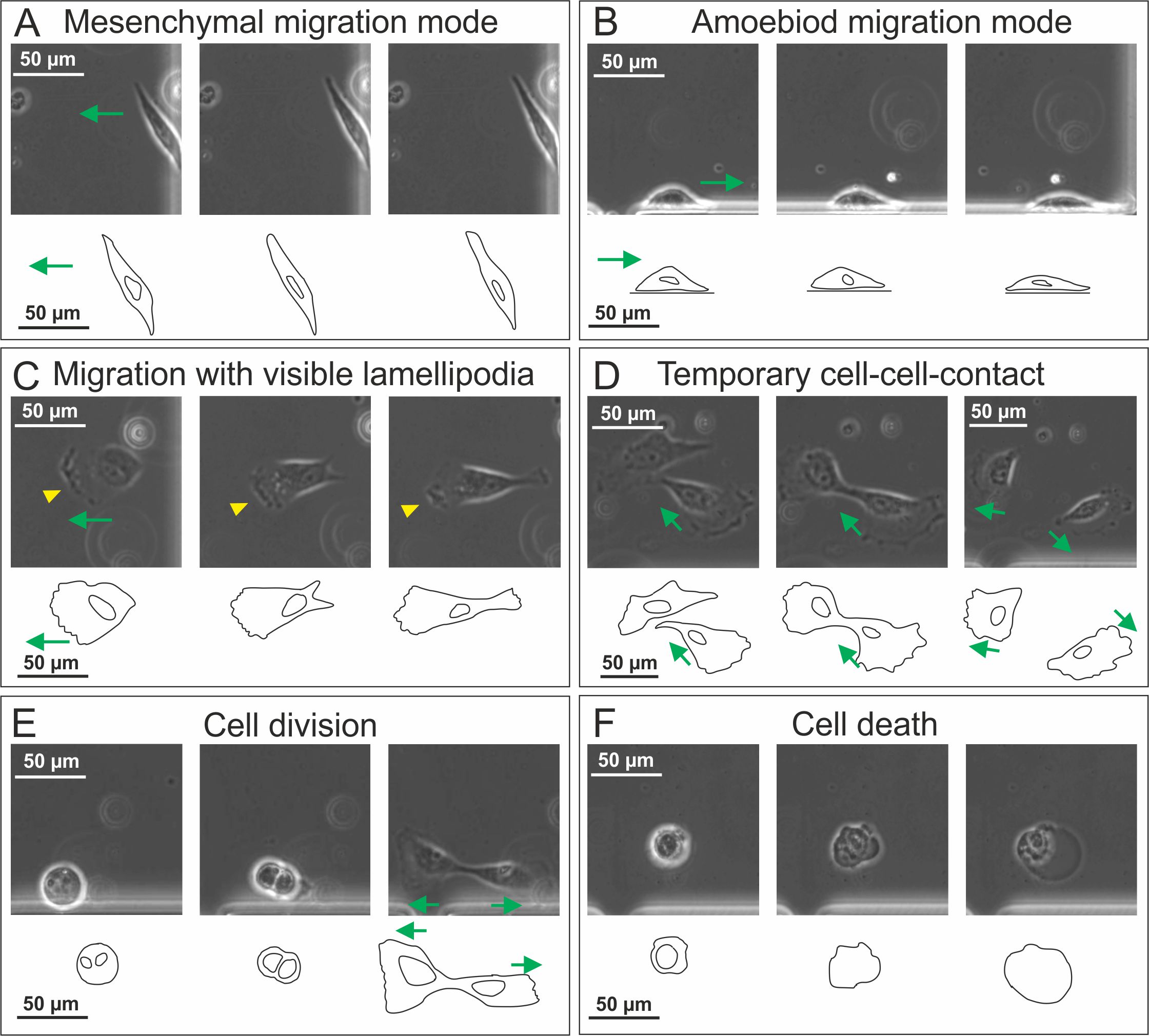

Supplement: Supplementary file 1 [file biology-10-00708-s001.zip › Supplemental Figure S1.jpg]

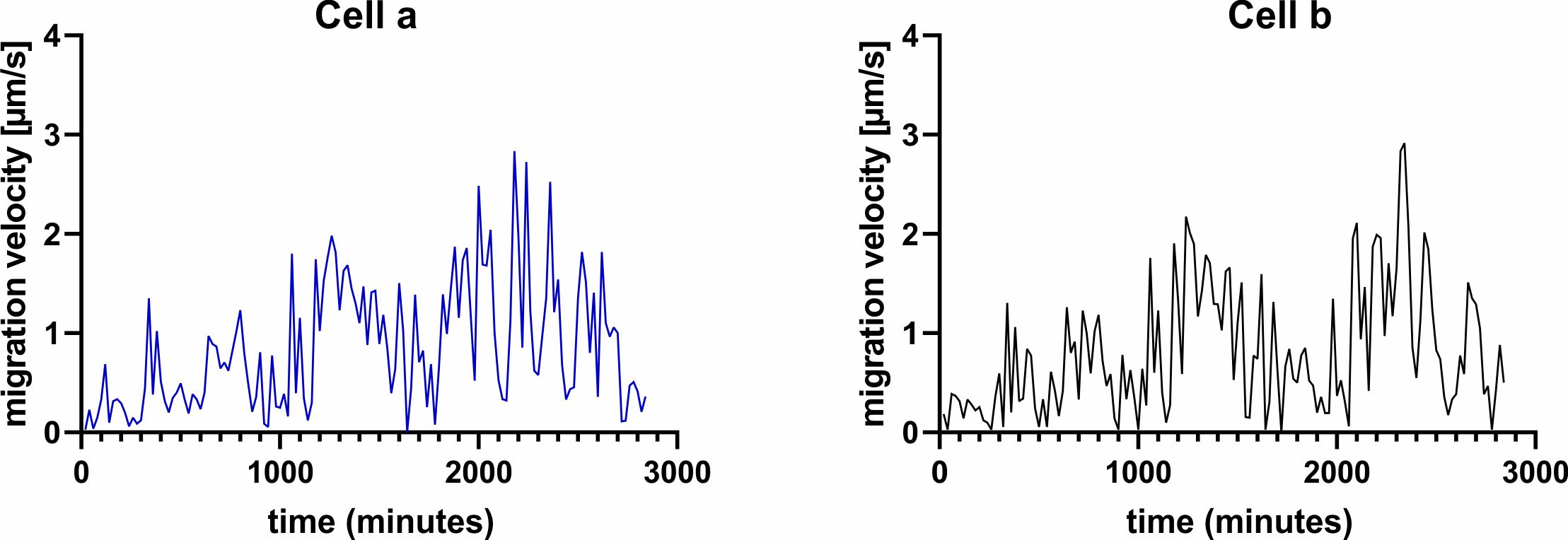

Supplement: Supplementary file 1 [file biology-10-00708-s001.zip › Supplemental Figure S2.jpg]

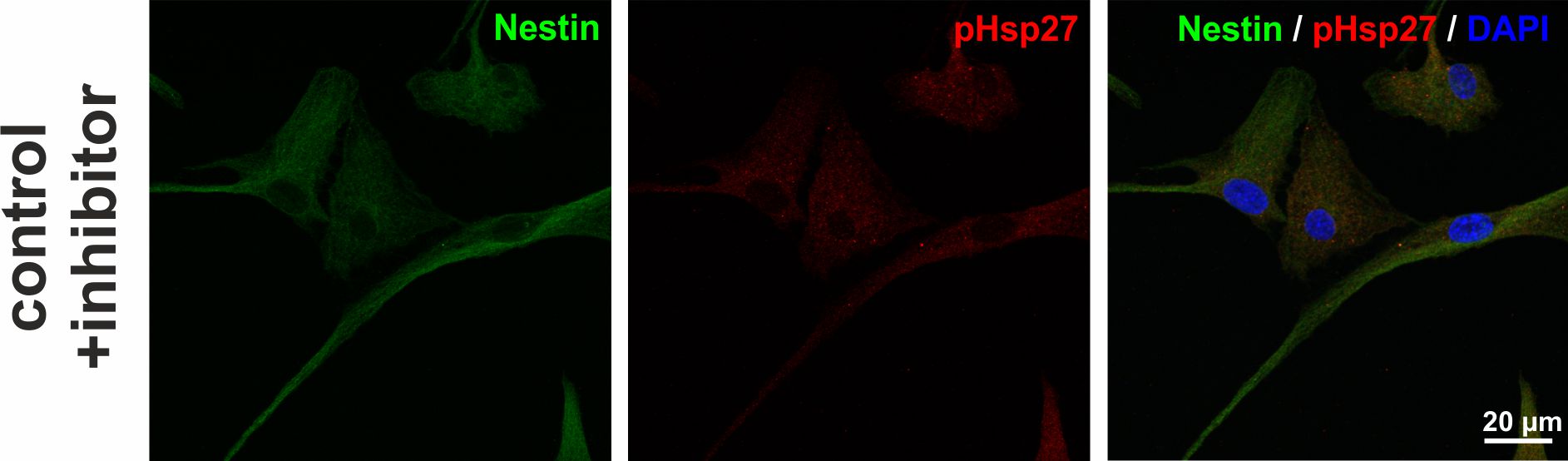

Supplement: Supplementary file 1 [file biology-10-00708-s001.zip › Supplemental Figure S3.jpg]
